# Molecular Determinants of Soft Tissue Sarcoma Immunity: Targets for Immune Intervention

**DOI:** 10.3390/ijms22147518

**Published:** 2021-07-13

**Authors:** Marcella Tazzari, Laura Bergamaschi, Alessandro De Vita, Paola Collini, Marta Barisella, Alessia Bertolotti, Toni Ibrahim, Sandro Pasquali, Chiara Castelli, Viviana Vallacchi

**Affiliations:** 1 Immunotherapy-Cell Therapy and Biobank Unit, IRCCS Istituto Romagnolo per lo Studio dei Tumori (IRST) “Dino Amadori”, 47014 Meldola, Italy; marcella.tazzari@irst.emr.it; 2 Unit of Immunotherapy of Human Tumors, Department of Research, Fondazione IRCCS Istituto Nazionale dei Tumori, 20133 Milan, Italy; laura.bergamaschi@istitutotumori.mi.it (L.B.); viviana.vallacchi@istitutotumori.mi.it (V.V.); 3 Osteoncology and Rare Tumors Center, IRCCS Istituto Romagnolo per lo Studio dei Tumori (IRST) “Dino Amadori”, 47014 Meldola, Italy; alessandro.devita@irst.emr.it (A.D.V.); toni.ibrahim@irst.emr.it (T.I.); 4 Department of Diagnostic Pathology and Laboratory Medicine, Fondazione IRCCS Istituto Nazionale dei Tumori, 20133 Milan, Italy; paola.collini@istitutotumori.mi.it (P.C.); marta.barisella@istitutotumori.mi.it (M.B.); alessia.bertolotti@istitutotumori.mi.it (A.B.); 5 Department of Surgery, Fondazione IRCCS Istituto Nazionale dei Tumori, 20133 Milan, Italy; sandro.pasquali@istitutotumori.mi.it

**Keywords:** soft tissue sarcoma, genetic landscape, immune profiling, tumor microenvironment, immunotherapy, targeted therapy, epigenetics, combination therapy

## Abstract

Soft tissue sarcomas (STSs) are a family of rare malignant tumors encompassing more than 80 histologies. Current therapies for metastatic STS, a condition that affects roughly half of patients, have limited efficacy, making innovative therapeutic strategies urgently needed. From a molecular point of view, STSs can be classified as translocation-related and those with a heavily rearranged genotype. Although only the latter display an increased mutational burden, molecular profiles suggestive of an “immune hot” tumor microenvironment are observed across STS histologies, and response to immunotherapy has been reported in both translocation-related and genetic complex STSs. These data reinforce the notion that immunity in STSs is multifaceted and influenced by both genetic and epigenetic determinants. Cumulative evidence indicates that a fine characterization of STSs at different levels is required to identify biomarkers predictive of immunotherapy response and to discover targetable pathways to switch on the immune sensitivity of “immune cold” tumors. In this review, we will summarize recent findings on the interplay between genetic landscape, molecular profiling and immunity in STSs. Immunological and molecular features will be discussed for their prognostic value in selected STS histologies. Finally, the local and systemic immunomodulatory effects of the targeted drugs imatinib and sunitinib will be discussed.

## 1. Introduction

The immune system serves as a safeguard against cancer development by eliminating cells harboring traits of genomic instability, such as unbalanced karyotype, copy number alterations and mutations in canonical drivers. Tumor genotype has also a direct effect on redirecting the host immune system [1]. The blueprint of this phenomenon is certainly represented by cutaneous melanoma, where mutations occurring in either BRAF or RAS oncogenes differently influence the host immunity at the tumor site [2]. The cumulative number of somatic mutations accounting for the tumor mutational burden (TMB; total number of mutations per mega base in tumor tissue) and translating into the neo-antigen load correlates with the tumor ability to evoke anti-cancer immunity and, indeed, response to immunotherapy [3]. However, from a Darwinian perspective, the genetic alterations endow tumor cells with the capability of evading immune destruction, which is recognized as a fundamental cancer hallmark [4]. Thus, in a favorable context for immune response, such as the case of tumors with high TMB and consequently a high yield of neo-antigens, cancer genotype evolves toward the acquisition of genetic determinants promoting immunosuppression.

Soft tissue sarcomas (STSs) include a variety of histological and molecular different rare malignant tumors with a wide range of differentiation towards mesenchymal lineages including muscle, adipose and other connective tissues. Genetically, STSs include tumors characterized by specific translocations which become driver of tumor pathogenesis and precise diagnostic markers, and tumors that display complex genomics alterations which include copy number alterations, mutations in canonical drivers and in general are characterized by a deep genetics and chromosomal instability.

STSs display a wide range of clinical behavior from low grade to high grade tumors that are characterized by an increased risk of metastatic spread. Surgery is the mainstay of treatment for localized disease, while systemic treatments, including chemotherapy or targeted therapy, are used for patients with metastasis. Recently, clinical trials tested immunotherapy strategies in STSs, showing effectiveness in selected histologies such as dedifferentiated liposarcoma (DDLPS) and undifferentiated pleomorphic sarcoma (UPS) [5]. However, results have been below expectations, likely reflecting the limited knowledge of STSs immune contexture, which is certainly hindered by the complex variety of STS histotypes and by their relative low incidence.

From an immunogenomic perspective, only sarcomas carrying complex karyotypes could selectively display an “immune hot”, heavily immune infiltrated tumor microenvironment (TME), making these tumors the only sarcomas likely to be responsive to immunotherapy. However, the epigenetic regulation of the transcriptional program, occurring at different levels, including DNA methylation, histone modification and chromatin remodeling, influences the tumor immune landscape [6]. For instance, in other solid cancers, such as melanoma and clear cell renal cell carcinoma, it has been shown that alterations of genes encoding modifiers of the SWI/SNF chromatin remodeling complex such as PBRM1, ARID2 and BRD7 could be associated with response to therapy with immune checkpoint inhibitors (ICIs), cellular-based vaccines or adoptive T cell therapies [7,8].

These epigenetic mechanisms influencing immunity are likely active in mesenchymal neoplasms, such as sarcomas, in which alterations in chromatin regulator genes are common [9]. For instance, a recent study has assessed tumor DNA methylation profiling as a novel candidate biomarker to select sarcoma patients who would potentially benefit from anti-PD-1 ICI therapy [10]. These additional insights might support the presence of “immune hot” TME also across histologies characterized by a translocation and not only among sarcomas carrying a complex genotype.

Nonetheless, some variations in the immune properties of each tumor can be heritable and determined by the germline genotype of the host, adding a level of complexity in the molecular determination of TME composition and functional orientation [11,12].

Compared to other tumors, characterization of immune infiltrate in sarcomas is in its infancy and a systematic exploration of correlations between tumor genetic and immune signatures, that also account for the distinctive features of each sarcoma histotype, is needed. The present review aims at picturing the complexity of sarcoma immunity and discussing genetic and epigenetic mechanisms that influence the sarcoma immune contexture.

## 2. Soft Tissue Sarcoma Classification: A Matter in Continue Evolution

STSs are a heterogenous group of mesenchymal tumors. The 2020 WHO classification of Soft Tissue and Bone Tumors [13] clearly described the complexity of this family of rare cancers by grouping STSs according to their line of differentiation, including adipocytic, fibroblast/myofibroblastic, the so-called “fibrohistiocytic”, vascular, pericytic, smooth and skeletal muscle, chondro-osseous and peripheral nerve sheath tumors. Gastrointestinal stromal tumors (GISTs) are included in this classification as well, together with tumors of uncertain differentiation. Undifferentiated small round cell sarcomas of bone and soft tissue, which includes Ewing sarcoma and other sarcomas characterized by various rearrangements, is a new group introduced in the 2020 WHO classification. Indeed, this last classification includes also new entities, such as myxoid pleomorphic liposarcoma, a myxoid liposarcoma with non-specific karyotype and lacking the classical gene fusion of DDIT3 with FUS or ESWR1 [13,14,15].

Great help in the definition of each single entity is nowadays provided by the identification of sarcoma histology-specific molecular alterations.

Sarcomas are classified into two main large groups, differing at the cytogenetic level. The group characterized by relatively simple karyotypes includes sarcomas with chromosomal translocations leading to fusion oncogenes (e.g., FUS/EWSR1-DDIT3 and PAX3/7-FOXO1, which characterize myxoid liposarcoma (MLS) and alveolar rhabdomyosarcoma (ARMS), respectively) and oncogene-mutated sarcomas (e.g., KIT, PDGFRA, RAS and BRAF mutations in GIST). Translocated sarcomas often affect children and young adults, people in whom tumorigenesis occurs from a small number of genetic events. Epigenetic mechanisms of gene regulation are involved in the majority of translocated sarcomas. FUS and EWSR1, which are the most common fusion partners in translocated fusion oncoproteins and characterize several sarcoma histologies, are drivers of the three-dimensional chromatin structure. An additional example of translocated sarcoma is synovial sarcoma (SS), which is among the common histologies localized in the extremity and superficial trunk and is characterized by a translocation which causes the fusion of SSX1 or 2 or 4 with SS18. The resulting chimeric oncoprotein is involved in chromatin remodeling [9].

The second group of STSs presents complex karyotypes, and are characterized by genomic instability. Sarcomas with a complex karyotype and non-reproducible genetic alterations usually occur in older patients who have accumulated numerous mutations or genetic deregulations, and they account for more than half of STSs. They are characterized by pleomorphic/spindle cell morphology and include pleomorphic/DDLPS, leiomiosarcoma (LMS) and pleomorphic leiomiosarcoma (PLPS), pleomorphic rabdomiosarcoma, malignant peripheral nerve sheath tumor (MPNST), myxofibrosarcoma (MFS), UPS and embryonal rhabdomyosarcoma (ERMS) for which complex karyotype is generally reported, although loss of heterozygosity of 11p15 region is found in most cases of ERMS. Among these tumors, UPS is the most frequently occurring sarcoma of extremity and trunk wall and has gained interest in the sarcoma community for being a polyclonal disease in which ICIs showed effectiveness in phase I and II clinical trials [16,17,18].

## 3. Studies on Genetic and Immunity of Sarcoma Are Tightly Connected

Advances in next-generation sequencing (NGS) technologies, such as whole-exome sequencing (WES) and whole-genome sequencing (WGS), are contributing to the discovery of novel genetic alterations within different sarcomas. The genomic landscape of each STS has helped re-classifying intra-histologic entities and also opened up avenues for targeted histology-specific treatment strategies. NGS approaches also uncover genetic alterations missed by conventional molecular diagnostic approaches, such as KIT/PDGFRA/SDH/RAS-P GIST, which was identified among wild-type GISTs [19,20]. These newly discovered alterations can potentially ensure eligibility for a specific molecular targeted treatment. Moreover, an unearthed oncogenic mutation might itself be a novel therapeutic target and its dysregulated expression/signaling might possess immunomodulating properties. Hence, oncogenic triggering could translate into the expression of druggable immune molecules or, more in general, pave the way for novel sequential or combined immune-mediated treatment [21].

Transcriptomic sequencing (e.g., bulk and single-cell RNA sequencing, Nanostring technology) and proteomic-based approaches (e.g., MS-CyTOF) integrated the tumor genetic landscape with the tumor immune profiling. In this context, NGS techniques have been applied to sarcoma to derive immune cell frequencies, the expression profile of immune-related genes, and also the T cell repertoire at the tumor site [22]. Intriguingly, while for long time sarcomas have been considered neglected by the host immune system, these omics approaches contributed to picture different scenarios of immune complexity at the TME level, not only across different histologies, but also within a single histology, reflecting the distinctive genetic and epigenetic background of STSs. The in silico query on gene expression public tumor datasets, such as The Cancer Genome Atlas (TCGA), together with the development of deconvolution methods able to retrieve immune cell components from mixture FFPE samples, have certainly sped up our understanding of immune cell composition across tumors and addressed unexpected immune contexture across sarcoma histologies [23,24,25].

**Figure 1:**
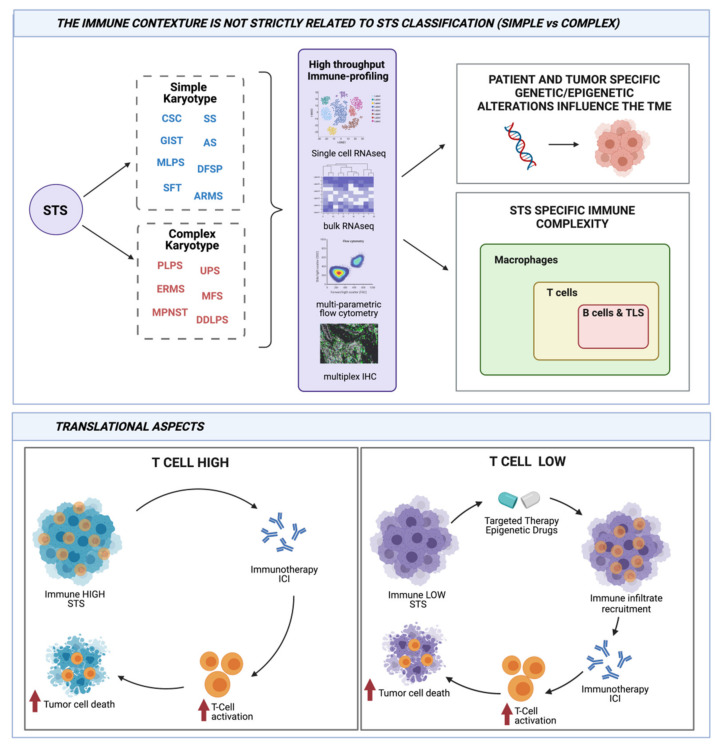
Connection between genetic and immunity in sarcomas and its translational relevance. STS dichotomization in simple and complex karyotypes does not reflect a sharp demarcation of immunogenicity and in situ immune infiltration. Along the characterization of patient- and tumor-related genetic alterations, the actual availability of high-throughput technologies has allowed immune profiling across and within distinct STS histologies. These analyses have highlighted previously unaddressed associations between genetic and/or epigenetic landscapes and peculiar immune contextures. From a translational point of view, while T cell-infiltrated STSs can benefit from immunotherapies, in T cell desert STSs, treatment by targeted or epigenetic drugs should be envisaged as therapeutic strategies to create a host environment potentially amenable to immunotherapies. Created with BioRender.com.

Besides the genetic approaches, immunohistochemistry (IHC) remains a fundamental technique to investigate the TME. The recent development of high-throughput multiplex IHC, able to dissect multiple markers on a single slide, and of related digital imaging analysis systems has certainly boosted the potentiality of this technique. Moreover, IHC is used as either a standalone approach or often applied to validate transcriptomic data, allowing analysis of protein expression on intact tissue sections. This gives crucial information on the co-expression profiles of different immune markers and on their reciprocal intra-tumoral cellular distribution [26,27]. These approaches have contributed to underline the association between specific pattern of immune infiltration and patient survival. In the next paragraphs, we will depict the current scenario of TME studies in complex and simple karyotype sarcomas and we will discuss how TME is influenced by tumor genetics, also within the same histological sarcoma subtype (Figure 1).

## 4. Genetic Immunity in STSs with Simple Karyotype

Sarcomas with simple karyotype are characterized by specific driving alterations, including oncogenic mutations and translocations that univocally identified a distinct sarcoma. Constitutive activation of oncogenic signaling pathways that are caused by mutations in oncogenes negatively impacts the immune TME by promoting intratumoral immune cell exclusion or favoring the recruitment of immunosuppressive cells [1,6]. This mechanism has been extensively described in melanoma, in which oncogenic signaling driven by mutated BRAF creates an immunosuppressive TME [28].

Studies addressing genetic mutational status, oncogenic pathway activation and their association with immunological features in sarcomas are in early stages. Among STSs, GIST represents the most investigated disease from this viewpoint. This tumor is driven by mutated oncogenes as it is characterized by gain of function mutations in the KIT or PDGFRA proto-oncogene. Although KIT- and PDGFRA-mutated GISTs display different clinicopathologic features, the mechanisms linking mutation to the different biological behaviors have not been understood yet. The majority of PDGFRA-mutant GISTs harbor D842V mutation in exon 18 and, more rarely, mutations in exons 12 and 14. Of note, D842V mutation is the one known to confer primary resistance to imatinib. Interestingly, recent data suggest that PDGFRA-mutant GISTs, and especially those with the D842V mutation, express higher levels of immune-related genes and are characterized by higher immunological activity compared to the KIT-mutated GISTs, or non-D842V mutant GISTs [29,30]. In general, PDGFRA mutation is associated with the over-expression of chemokines, including CXCL14, and of the immune checkpoint molecules BTLA, CD48, TNFRSF9 and TIGIT. Accordingly, PDGFRA-mutant GISTs were more likely to display intra-tumoral tertiary lymphoid structures (TLSs) [31]. Remarkably, the TME of PDGFRA-mutant GISTs is generally enriched in cytotoxic immune cells and an increased production of high-affinity neoepitopes has been documented. In addition, an enhanced production of HLA binders was observed in particular in D842V mutant GISTs [29]. Whether increased neoepitopes production and this “immune hot” profile may indeed account for the relatively favorable patient outcomes of PDGFRA-driven compared to KIT-mutated GIST remains an intriguing hypothesis deserving further investigations.

Part of the clinical success of the imatinib targeted therapy in KIT-mutated GIST likely also relies on immune-related effects involving both innate and adaptive immunity. In fact, it has been shown that in patients with gastrointestinal tumors, imatinib boosts the IFNα secretion by natural killer cells and concomitantly, it decreases regulatory T cell numbers by inducing their apoptotic death [32]. Imatinib also limits/blocks the production of IDO by GIST cells, thus alleviating tumor-induced immunosuppression in the TME and allowing reactivation of cytotoxic CD8+ T cells. In agreement with these data, in freshly obtained human GIST specimens, the T cell profile correlated with tumor response to imatinib sensitivity [33]. Moreover, in several human tumors, increasing evidence is now collected showing that kinase inhibitors foster parallel strategies by blocking the oncogenic signaling and affecting immunological contexture at different levels. For instance, we were able to document the immunomodulatory capacity of imatinib in the context of a rare sarcoma, dermatofibrosarcoma protuberans, as later discussed in this review [34,35].

A correlation between fingerprint mutations and immunity at the tumor site has also been recently reported for angiosarcomas, a rare group of sarcomas arising from endothelial cells of vessels that harbor mutations in angiogenic and oncogenic signaling pathways. These tumors are often characterized by multifocal lesions and poor outcomes. A recent study reported that a subset of angiosarcoma overexpresses at transcriptional level inflammation- and immune-related signaling pathway genes and is characterized by a high number of neutrophils (CD15+), macrophages (CD68+), cytotoxic T (CD8+), Tregs (FOXP3+) and PD-L1+ immune cells. Immune inflammation signatures were enriched in two mutually exclusive angiosarcoma groups, one harboring UV-related signature (and thus displaying high TMB) and the second positive for human herpes virus-7 (HHV-7). HHV-7 virus might directly drive inflammation-related pathways, including IFN response genes and, conversely, evoke immune evasion, finally leading to an exhausted TME [36]. Clearly, the integration of genomic, transcriptomic and immune contexture data defines discrete subtypes of angiosarcomas, opening up novel avenues for improving prognostic risk stratification and, ultimately, treatment for this rare STS.

Epigenetic dysregulation can be induced at different levels, which include alterations in DNA methylation patterns, histone mutations and modifications, alterations in chromatin remodeling complexes and in 3D chromatin structure [9,37]. Translocation-driven sarcomas are characterized by fusion proteins acting as epigenetic modulators, inducing dysregulation of transcriptional programs of downstream genes, ultimately resulting in altered phenotypes. The most common fusion-driven sarcomas include synovial sarcoma (SS), fusion-positive alveolar rhabdomyosarcoma (ARMS) and myxoid liposarcoma (MLS). SS is characterized by the fusion oncoprotein SSX-SS18. Rhabdoid tumors are pediatric cancers driven by loss of SMARCB1/INI1. Both genetic alterations result in an aberrant activity of SWI/SNF family of multiprotein complexes, crucial effectors of chromatin remodeling. ARMS and MLS carry gene fusions involving transcription factors. ARMS usually harbor translocations fusing FOXO1 to PAX3 or PAX7 genes, while MLS are characterized by a translocation involving CHOP—also known as DDIT3—and FUS, or, at lower frequency, EWSR1 genes [9,38,39,40].

Unfortunately, the majority of fusion oncoproteins cannot be considered actionable targets for therapeutic intervention and alternative therapeutic approaches aimed at targeting epigenetic dysregulated factors have been considered. Sarcomas, such as SS and ARMS, characterized by the disruption of the SWI/SNF chromatin modifier complex, show overactivation of methyltransferase EZH2, which can be targeted by Tazemetostat [38]. Importantly, dysregulation of epigenetic mechanisms is common also in STSs with complex karyotype. An example is represented by epithelioid sarcoma displaying loss of INI1, a chromatin modifier belonging to the SWI/SNF family. Indeed, EZH2 inhibitor has been recently approved by the FDA for this sarcoma [41,42].

However, in general, a deeper characterization of mechanisms of action of epigenetic drugs and their off-target effects is needed and is mandatory to optimize their use in clinical settings. From this perspective, as in other tumors, such as melanoma and clear cell renal cell carcinoma, alterations of genes encoding modifiers of the SWI/SNF chromatin remodeling are associated with response to ICIs [7,8]. However, the connection between the immunological landscape and the epigenetic program in the majority of translocation-driven STSs is a completely unexplored field deserving further attention.

An initial attempt in this direction has been recently pursued for SS. Indeed, SS fusion oncoprotein SS18–SSX inhibits cell differentiation, sustains cell cycle progression and is negatively associated with immune infiltrate. Treatment of SS cells with epigenetic drugs targeting HDAC and modulators of the cell cycle represses the oncogenic program, increases tumor cell immunogenicity and, importantly, oncogenic program can be suppressed by cytokines released by macrophages and T cells within the TME [43]. Taken together, these data highlight a strong bidirectional cross-talk between immunity and oncogenic programs in SS and the possible synergistic actions of drugs directed against oncogenic program and immunotherapy. Moreover, it stresses the need to further investigate the relationship between epigenetic change, activation and immune response to identify new potential therapeutic approaches for translocated sarcomas. Of note, this relationship should be also explored in sarcomas with complex karyiotypes, as an association between SWI/SNF chromatin remodeling alteration and immunity has recently been described in pleomorphic sarcomas [42].

## 5. Genetic Immunity in STSs with Complex Karyotype

In sarcoma, mirroring other largely investigated tumors, genomic complexity translates to increased TMB and immune infiltrates. For instance, UPS, which is the most frequent STS arising in the extremities and superficial trunk, and with a high risk of disease progression, is characterized by complex karyotype and the lack of a specific line of histological differentiation [44]. UPS displays an “immune hot” TME that correlates with clinical and pathologic features. Wustrack and colleagues uncovered an inverse correlation between tumor-infiltrating CD8-positive T cells and tumor size and, conversely, a direct correlation between CD8 T cells and better patient survival [45]. This observation is in line with findings from clinical trials, reporting that patients with UPS have the highest chance among sarcoma patients of developing a tumor response to immunotherapy with ICIs in metastatic setting [5,46]. Moreover, a recent study identified two main disease entities of UPS, characterized by different patient survival, magnetic resonance imaging (MRI) textures and molecular features. The group from patients with better survival was strongly enriched in genes involved in immunity [47]. This comprehensive UPS omics characterization provide the rationale for selecting the best candidate tumors for immunotherapy within the same histotype, and also has additional important implications from a translation perspective.

The group of UPS lacking immune infiltrate was enriched in genes involved in tumor development and stemness, and in particular, it displayed an up-regulation of PI3K/AKT and Wnt/beta catenin pathways, which are well known to be associated with immune exclusion in many tumors, including other sarcomas [48,49]. Preclinical studies suggested that dysregulation of the stemness-related FGFR2 signaling pathway plays a key role in the oncogenic processes of the immune-desert UPS and FGFR2 pharmacological inhibition impaired the tumor growth of patient derived xenograft. The direct interplay between the oncogenic process and immune evasion found in SS is likely occurring in UPS, as well. Whether blocking the FGFR2 pathway also restores the immune sensitivity of immune-desert UPS remains to be explored. Certainly, addressing this question might inform the design of treatment strategies that combined targeted therapies and immune anti-cancer agents.

The transcriptomic profiling analysis by the TCGA consortium revealed that UPS, MFS and DDLPS display a high macrophage score, regardless of T cell infiltration. Additionally, in UPS and MFS, the presence of dendritic cells and immature dendritic cells correlates with better survival, while enrichment of Th2 signature in DDLPS, although expressing the highest CD8 T cell score, correlates with shorter survival. Of note, these sarcoma histologies were marked by a high expression of druggable immune markers B7-H3, TGFB1 and TIM3 [25]. Furthermore, WES analyses have deepened the genetic and epigenetic landscape of MFS, showing three MFS subtypes with distinct DNA methylation signatures. Integrated analyses have shown a correlation between these clusters and differences in immune cell compositions. In particular, CD8+ T cell fraction was significantly higher in the cluster characterized by better survival, suggesting a prognostic value for CD8+ T cell infiltration in MFS [50].

The presence of distinct phenotypes, according to the immune composition of the TME in DDLPS, MFS and UPS, was further confirmed in two additional studies [51,52]. In situ analysis, conducted by multiplex IHC, showed that immune infiltrate organized in TLSs, consisting of B, T and follicular dendritic cells, was tightly associated with response to immunotherapy and better patient outcomes [52]. In another study, TLSs were distinctive traits also in ARMS and ERMS, which are characterized by a less complex karyotype compared to UPS, MFS and DDLPS. Interestingly, this work included samples from patients diagnosed with UPS and RMS, two STS subtypes showing opposite response to ICIs [53]. IHC comparisons of their TME revealed a similar immune niche that was dependent on angiogenesis and constituted mainly by myeloid cells, with UPS showing the highest infiltration compared to ERMS, followed by ARMS. Interestingly, these histologies differed in the spatial distribution of T cells, with UPS being characterized by diffuse infiltration of T cells and RMS marked by T cells only clustered in TLSs. Abundance of macrophages in RMS was also highlighted by a recent study showing that the microvascular density correlates with immune cell infiltration and that both CD163-positive macrophages and CD54-positive microvessels were more likely detected in ERMS than ARMS and correlated with patients’ overall and event-free survival [54].

## 6. Translational Aspects of Genetic Immunity Interaction

Targeted therapy drugs belonging to the anti-angiogenic family, such as sunitinib and pazopanib, have been used for different STS histologies, including solitary fibrous tumors (SFT) and clear cell sarcomas (CSC). In these simple karyotype STSs, we have shown that these drugs modulate systemic and local host immune components [55,56,57] and convert the immune TME from “cold” to “hot”. Indeed, sunitinib, by virtue of its effect of the tumor vasculature, promoted an influx of T cells within the previously T cell desert tumor bed. Moreover, it down modulated the frequency of circulating immune suppressive cells, regulatory T cells and myeloid-derived suppressor cells, thus unleashing the adaptive anti-tumor immune response [55]. Of note, in CSC, a very rare sarcoma that shares clinical, pathological and features typical of cutaneous melanoma, it was observed that such a response was antigen-specific towards melanoma-associated antigens [56]. Our data reinforce the notion that treatment with targeted therapy drugs modulates host immunity in simple karyotype sarcomas, which are generally characterized by an inherent low level of local and systemic anti-tumor immune response. Indeed, after treatment with targeted therapies, these histotypes could become potentially sensitive to immunotherapy [39]. A clear connection between angiogenesis and adaptive immunity is demonstrated and normalization of the tumor vascularization with antiangiogenic drugs will be helpful in favoring T cell tumor trafficking and limiting immunosuppression mechanisms active at tumor site. A combination of antiangiogenic drugs with ICIs is under study in ongoing or recently closed clinical trials for several sarcomas, including alveolar soft part sarcoma, angiosarcoma, SFT and UPS [58,59].

**Figure 2:**
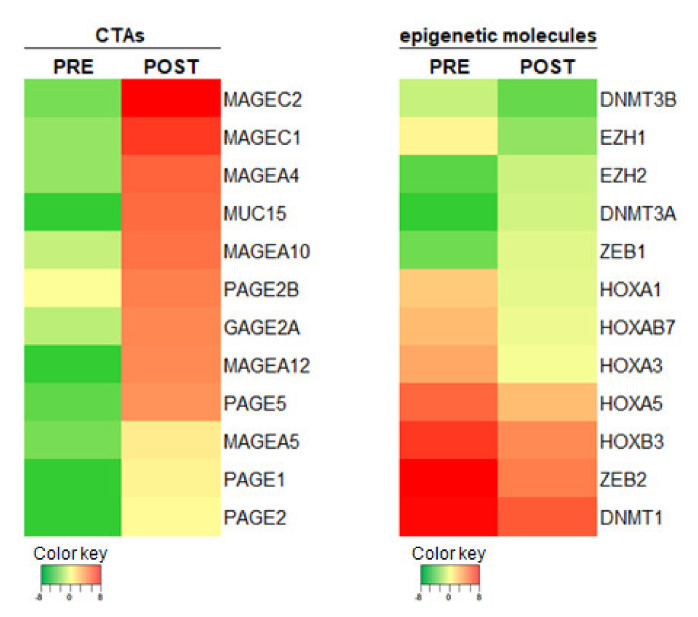
Genes modulated by imatinib in FS-DFSP. Heatmaps showing the expression levels of selected genes encoding cancer testing antigens (CTAs) or epigenetic molecules determined by RNAseq analysis of FS-DFSP pre- and post-imatinib treatment.

Adaptive immunity was boosted and found to be crucial also in the therapeutic responses to imatinib of fibrosarcomatous DFSP (FS-DFSP), a variant of DFSP characterized by occurrence of lung metastasis, an event that does not occur in pure DFSP [35,60]. Intriguingly, besides the observed modulation of the immune contexture and the induced tumoral PD-L1 upregulation as a potential immune escape mechanism dampening the imatinib-induced anti-tumor response, no direct immunomodulating effects on the tumor cells were identified. Unpublished results obtained reanalyzing autologous pre- and post-imatinib RNA sequencing data, available from a single patient, are indicative of an epigenetic direct effect of this drug in modulating tumor cell immunogenicity. Dysregulation of genes associated to epigenetic modifications and upregulation of a set of genes encoding for cancer testis antigens (CTAs) was observed in post-treatment tumors (Figure 2). We may speculate that CTA upregulation, together with the reported elevated expression of HLA class I, acts as actionable antigens for adoptively transfer of antigen-specific T cells.

These data are in line with increasing evidence indicating that epigenetic processes have a key role in the regulation of immune cell activity and anti-tumor response. Particularly, molecular events disrupting epigenetic balance at the tumor level could promote the escape of tumor cells from immune surveillance, mainly by down-regulating the expression of tumor-associated antigens, antigen-presenting machinery and co-stimulatory molecules [61].

Interfering with epigenetic mechanisms also has a direct effect on immune cell subsets. For example, EZH2 targeting reprograms the functional activity of intratumoral regulatory T cells and activates and influences T cell differentiation and function [62,63,64]. Results of these studies provided the rationale to combine epigenetic drugs and immunotherapy to reshape TME and increase anti-tumor immune response in sarcomas. For instance, this experimental therapeutic approach, using the EZH2 inhibitor Tazemetostat in combination with Durvalumab, a PD-L1 inhibitor, is under investigation in clinical trials for solid tumors, including sarcomas (NCT04705818).

It has been recently reported that aberrant activation of cell cycle in tumor cells promotes immune evasion. Genomic and transcriptomic studies revealed that amplification of cyclin D1 and CDK4 genes are associated with resistance to immunotherapy and that tumors with hyperexpression of genes promoting cell cycle display a “cold” immune tumor contexture. In addition, recent evidence demonstrates that treatment of tumors with CDK4/CDK6 inhibitors could restore regulatory mechanisms, including regulation of IFNγ signaling, antigen presentation machinery and cell release of immunomodulating factors, leading to improve the immune anti-tumor activity [65]. Indeed, CDK pathway activation is now recognized as a driver of sarcomagenesis and CDKs are emerging therapeutic targets for different types of sarcomas [66]. CDK4/6 inhibitors in combination with ICIs are currently underway in phase I–II clinical trials for selected solid tumors, including head and neck squamous cell carcinoma and hepatocellular carcinoma (NCT03655444, NCT03781960). We observed that patients affected by myxoid liposarcomas and with poor prognosis are characterized by the hyperexpression of genes associated with cell cycle activation and proliferation, and that hyperexpression of cyclins tightly correlated with low expression of immune-related genes [unpublished data]. Altogether, these observations lead us to hypothesize whether a combination therapy with cyclin inhibitors and immunotherapy should be considered a treatment option in selected sarcoma histologies.

## 7. Genetic/Immunological Diversity in STSs and Response to ICIs

ICIs have substantially improved prognosis for several solid cancers, including malignant melanoma, lung, renal, urothelial and head and neck cancer. In STSs, the clinical benefit of ICIs showed controversial and in general unsatisfactory results. As outlined in this review, STSs include different histological types with different biological and consequently immunological features. Most importantly, each single histotype displays genetic variations that directly affect the TME composition. Thus, this large variety together with the relative low frequency of these rare tumors, certainly limit the possibility to clearly interpret the efficacy of ICI therapy in STSs and the development of predictive and reliable biomarkers to define which sarcoma patients are likely responsive to ICIs. To overcome these limits, systematic omics studies of STSs should be planned. In particular, transcriptional studies at single cell level should be performed in order to determine how tumor and non-tumor cells could promote the onset of resistance.

A recent meta-analysis showed that high response rates to ICIs are found—besides in the classical Kaposi sarcoma, also in alveolar soft tissue sarcoma (ASPS) and in UPS, showing an ORR of 0.35 and 0.20, respectively [67]. This result supports the conclusion that not only sarcomas with complex karyotypes and displaying high TMB can be targets for ICIs, but also translocation-driven sarcomas are susceptible to ICI-based immunotherapy. In translocation-driven sarcomas, epigenetic regulation of the transcriptional program might induce the tumor immune landscape to become permissive to ICI response. The finding that still a limited fraction of patients in each histological sarcoma subtype experiences a clinical benefit from ICI treatment may reflect the genetic and immunological heterogeneity that dominate each single histology and that we have illustrated for UPS, angiosarcomas and GIST. Data showing the genetic and immunological diversity inside the ASPS are not currently available, but, taken together, all these data highlight the crucial importance to deeply investigate the molecular features linked to immune evasion to select the candidate tumors for immunotherapy also within the same histotype.

An additional important conclusion of this meta-analysis study is that early incorporation of ICIs with chemotherapy or with TKI shows improved ORR rates in sarcoma patients, further stressing the value of the combination of ICIs with drugs endowed with immunomodulating properties. Chemotherapy in STSs is mainly based on the usage of antracycline, whose activities in inducing immunogenic cell death is well documented [68]. Conversely the role of the anti-angiogenic drug in inducing a “hot” TME in sarcomas has been extensively discussed in the previous paragraph.

On the basis of results obtained in other tumors, clearly indicating that non-immune pathways could impact response to ICI therapy, an additional combination therapy that can be proposed for STS will envisage the use of drugs targeting CDKs [69]. In fact, CDK activation pathways are drivers of sarcomagenesis, but they also have an active role in limiting immune surveillance in human tumors and likely in sarcomas, as well. Moreover, it has been recently reported that CDK4/6 axis is a tumor-intrinsic resistance mechanism to anti PD1 therapy in melanoma [70] and increased activity of the CDK4/6 inhibitor CDKN2A correlates with response to PD-L1 blockage in RCC and NSCLC patients [69].

ICI response is strongly affected by the complex interplay between cancer cells and TME. A deep and systematic investigation of the immune niche of STSs is therefore mandatory to identify mechanisms promoting an immunosuppressive TME. Recent studies reported that in the majority of STSs, regardless of the histology and genetics, macrophages represent the dominant immune cell population, as they outnumber lymphocytes, and have a M2 pro-tumorigenic phenotype [53,71]. In addition, recent findings showed that STS-associated macrophages express high levels of IDO1, an enzyme involved in the production of kynurenine, a tryptophan metabolite that can promote the expansion of regulatory T cells [72]. Taken together, these data suggest a key role for these cells in sustaining an immunosuppressive TME, thus promoting resistance to ICI therapy. Combining ICIs with antimyeloid or metabolic reprogramming drugs, aimed at selectively eliminating this immunosuppressive component of STS TME, could therefore represent a promising therapeutic strategy for sarcoma patients.

## 8. Concluding Remarks

This review addressed the interconnection between genomic characteristics and immune response in different sarcoma histologies (Table 1). We highlighted the fine dissection of molecular mechanisms linking genetic and epigenetic alterations to immunomodulating events, particularly at the TME level. Overall, this review provides at least three clinically relevant observations. Firstly, the classification into complex and simple karyotype sarcomas does not necessarily translate into a sharp separation between “immune hot” and “immune cold” TME, as within complex sarcomas, which are recognized for their richest immune contexture, immunological characterization could lead to better selection of candidates to immunotherapy. Secondly, even if simple karyotype sarcomas are usually characterized by low TMB, their alterations at genomic levels and in epigenetic regulation of transcriptome deeply affect host antitumor immune response resulting in potential susceptibility to immunotherapy. Thirdly, treatment with targeted therapy or with epigenetic drugs may uncover new immune-related actionable determinants that could then be exploited therapeutically.

Several IHC-based studies of STS TME clearly documented an abundant myeloid cell infiltration spanning across sarcoma subtypes despite the simple and complex karyotype dichotomization. However, intra-tumoral myeloid composition and its relationship between active tumoral epigenetic and oncogenic programs is still an unexplored field. The precise role of these cells in tumor promotion and immunosuppression has been far less understood and advanced sequencing approaches, such as single cell RNA sequencing, should be applied to decipher their phenotypic and functional specifications. This could be translated into the identification of myeloid cells-based biomarkers that modify tumor response to immunotherapy as well as novel actionable targets, with the ultimate aim of providing sarcoma patients with effective treatment options that impact survival.

## Figures and Tables

**Table 1:** Studies connecting genetic features and immunity in selected sarcoma histotypes.

Sarcoma Subtype	Alteration	Hot Immune Landscape Associated To	Refs.
GIST	Oncogenic mutation	PDGFRA gene mutation	[29]
Angiosarcoma	Oncogenic mutation	UV signature and HHV-7 genome	[36]
SS	Fusion-driven	Downregulation of epigenetic and oncogenic program	[43]
MFS	Complex karyotype	Methylation pattern	[50]
UPS	Complex karyotype	Downregulation of stemness and FGFR2 signaling genes	[47]
Basket STS	Simple and complex karyotype	Methylation pattern	[10]

GIST: gastrointestinal stromal tumor; SS: synovial sarcoma; MFS: myxofibrosarcoma; UPS: undifferentiated pleomorphic sarcoma; STS: soft tissue sarcoma.

## Data Availability

The raw data that support findings of Figure 2 are available upon request.
